# The Effects of Viral Load Burden on Pregnancy Loss among HIV-Infected Women in the United States

**DOI:** 10.1155/2015/362357

**Published:** 2015-10-25

**Authors:** Jordan E. Cates, Daniel Westreich, Andrew Edmonds, Rodney L. Wright, Howard Minkoff, Christine Colie, Ruth M. Greenblatt, Helen E. Cejtin, Roksana Karim, Lisa B. Haddad, Mirjam-Colette Kempf, Elizabeth T. Golub, Adaora A. Adimora

**Affiliations:** ^1^Department of Epidemiology, The University of North Carolina at Chapel Hill, Chapel Hill, NC 27599, USA; ^2^Department of Obstetrics & Gynecology and Women's Health, Albert Einstein College of Medicine, Montefiore Medical Center, Bronx, NY 10467, USA; ^3^Department of Obstetrics & Gynecology, Maimonides Medical Center, Brooklyn, NY 11219, USA; ^4^Department of Obstetrics & Gynecology, Georgetown University, Washington, DC 20007, USA; ^5^Departments of Clinical Pharmacy, Medicine Epidemiology and Biostatistics, University of California, San Francisco, San Francisco, CA 94143, USA; ^6^Department of Obstetrics and Gynecology, John H. Stroger Hospital of Cook County, Chicago, IL 60612, USA; ^7^Department of Preventive Medicine, Keck School of Medicine, University of Southern California, Los Angeles, CA 90032, USA; ^8^Department of Gynecology and Obstetrics, Emory University School of Medicine, Atlanta, GA 30322, USA; ^9^Schools of Nursing and Public Health, Department of Health Behavior, University of Alabama at Birmingham, Birmingham, AL 35294, USA; ^10^Department of Epidemiology, Johns Hopkins Bloomberg School of Public Health, Baltimore, MD 21205, USA; ^11^School of Medicine, The University of North Carolina at Chapel Hill, Chapel Hill, NC 27516, USA

## Abstract

*Background*. To evaluate the effects of HIV viral load, measured cross-sectionally and cumulatively, on the risk of miscarriage or stillbirth (pregnancy loss) among HIV-infected women enrolled in the Women's Interagency HIV Study between 1994 and 2013. *Methods*. We assessed three exposures: most recent viral load measure before the pregnancy ended, log_10_ copy-years viremia from initiation of antiretroviral therapy (ART) to conception, and log_10_ copy-years viremia in the two years before conception. *Results*. The risk of pregnancy loss for those with log_10_ viral load >4.00 before pregnancy ended was 1.59 (95% confidence interval (CI): 0.99, 2.56) times as high as the risk for women whose log_10_ viral load was ≤1.60. There was not a meaningful impact of log_10_ copy-years viremia since ART or log_10_ copy-years viremia in the two years before conception on pregnancy loss (adjusted risk ratios (aRRs): 0.80 (95% CI: 0.69, 0.92) and 1.00 (95% CI: 0.90, 1.11), resp.). *Conclusions*. Cumulative viral load burden does not appear to be an informative measure for pregnancy loss risk, but the extent of HIV replication during pregnancy, as represented by plasma HIV RNA viral load, predicted loss versus live birth in this ethnically diverse cohort of HIV-infected US women.

## 1. Introduction

In 2013, women accounted for over half of the 35 million people living with human immunodeficiency virus (HIV) worldwide [1]. HIV-infected women who adhere to antiretroviral therapy (ART) are living longer, healthier lives [2, 3]. Due to improved maternal health and the low probability of mother-to-child transmission with effective and timely treatment [3–5], increasing numbers of HIV-infected women of reproductive age are deciding to become pregnant or expressing a desire for future childbearing [4–9].

Prior to the era of effective ART, HIV-infected women had a substantially higher risk of adverse birth outcomes [10–13], with one meta-analysis reporting that HIV-infected women had a risk of miscarriage or stillbirth four times as high as uninfected women [10]. ART has reduced but not removed this disparity in risk. While ART is effective at lowering viremia [3], several studies have shown that maternal HIV infection is associated with an increased risk of adverse birth outcomes even in the presence of ART [13–16], although not all studies have observed this association [17].

The pathogenesis of adverse birth outcomes, including the possible increased risk among HIV-infected women, is not well understood, though some mechanisms have been suggested. Successful live childbirth is a culmination of an inflammatory cascade, and it has been speculated that dysregulation of this cascade by viral antigens may lead to adverse birth outcomes [18–20]. Higher plasma viral load is a marker of maternal HIV disease severity and has been associated with adverse birth outcomes [16, 21, 22]. For example, in a study of African women, each log_10_ increase in maternal HIV RNA viral load measured during early pregnancy was associated with a 90% relative increase in the odds of stillbirth [21].

There are several limitations regarding the current literature on pregnancy loss in HIV-infected women. To date, no studies have investigated whether long-term immune system inflammation due to continuous viral load burden could have an effect on adverse pregnancy outcomes. One novel measure for quantifying cumulative viral load is copy-years viremia [22]. Copy-years viremia since ART initiation has been shown to be a predictor of mortality [23–25] and specific types of lymphoma among HIV-infected individuals [26]. Additionally, most studies are from low resource environments [14–16, 21, 22], with limited data addressing the impact of viremia on pregnancy outcomes in high resource settings [17]. Also, while there are some studies concerning the effect of viral load burden on infant outcomes, such as preterm birth and low birth weight, there are fewer and more contradictory studies assessing its relationship with pregnancy loss (miscarriage and stillbirth) [14–17, 21, 22].

To better understand the pathogenesis of pregnancy loss among HIV-infected women in the United States, we used three different specifications of viral load burden. Specifically, we assessed whether (1) a single measure of most recent viral load before the pregnancy ended, (2) cumulative viral load (log_10_ copy-years viremia from ART initiation to conception), or (3) short-term viral load (log_10_ copy-years in the two years preceding pregnancy) in HIV-infected women was associated with increased risks of pregnancy loss (miscarriage and stillbirth).

## 2. Methods

### 2.1. Study Population and Definitions

The Women's Interagency HIV Study (WIHS) is an ongoing, multicenter, prospective cohort study of HIV-uninfected and HIV-infected women enrolled across the United States. The WIHS, which includes a semiannual medical exam and interview, has been described in detail previously [27, 28]. Written informed consent was obtained after IRB approval of WIHS protocols at all participating institutions.

**Figure 1:**
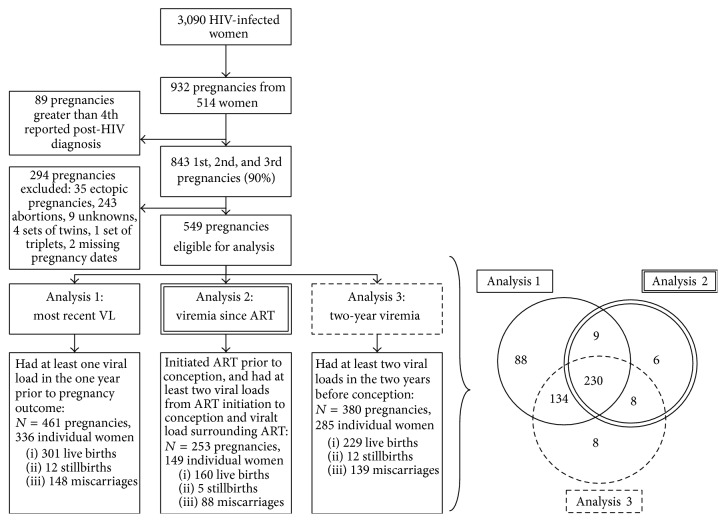
Flowchart and Venn diagram of women enrolled in the Women's Interagency HIV Study (WIHS) since 1994, illustrating the inclusion criteria for pregnancies included in analyses 1, 2, and 3. The Venn diagram illustrates the overlap between the three analytic populations.

To assess our three exposures, we constructed three separate analytic populations from the WIHS population (Figure 1). For all three populations, we analyzed data from 514 HIV-infected women enrolled in the WIHS, who provided data between October 1, 1994, and March 31, 2013, with at least one self-reported singleton pregnancy following enrollment. Only first, second, and third reported pregnancies (90% of all pregnancies) were included to reduce possible confounding by extreme gravidity. We excluded pregnancies without at least one viral load measure within the year prior to pregnancy outcome (analysis 1) and without at least two viral load measures prior to conception (analyses 2 and 3) (Figure 1). Analyses 1 and 3 included both ART-naïve and ART-initiated women, while analysis 2 was restricted to ART initiators as it focused on viral load since ART initiation.

ART was defined as use of any antiretroviral drug but was categorized as mono-/dual therapy and highly active ART (HAART). In a sensitivity analysis, we repeated analysis 2 but restricted it to those who initiated HAART. HAART was defined according to the US Department of Health and Human Services/Kaiser Panel guidelines [29].

Pregnancies and pregnancy outcomes were self-reported. Participants were asked at every visit, “have you been pregnant since your last visit?” (yes or no) and “what was the outcome of the pregnancy?” Pregnancy outcomes were explained to study subjects by trained interviewers to help reduce errors: miscarriage was defined as the spontaneous loss of a pregnancy before 20 weeks/5 months of gestation, stillbirth was defined as a child born dead after that time, and live birth was defined as a baby born alive [28]. The main outcome of interest, pregnancy loss, was defined as either a self-reported miscarriage or stillbirth and was compared to live births. Ectopic pregnancies were infrequent and were therefore excluded, while elective abortions were considered to be a presumptively uninformative competing risk and were therefore also excluded (Figure 1).

For pregnancies in which the woman had a WIHS visit during the pregnancy, we estimated that her date of conception was two weeks after her last menstrual period [30]. For all other pregnancies, assumptions based on published literature were used to estimate the date of conception: the approximate date of conception was estimated as 38 weeks before a live birth, 32 weeks before a stillbirth, and 11 weeks before a miscarriage [31–33].

HIV RNA viral load (copies per milliliter of plasma) was ascertained at each semiannual study visit. There were two challenges regarding the lower limit of detection (LLD) for the viral load assays used. First, assays changed over time with differing limits of detection; secondly, the viral loads at the LLD cannot be assumed to truly be at this left-censored measurement limit. For example, the LLD for the most commonly used assay during the full study period was 80: a woman with a viral load of 80 was assumed to have a true viral load between 0 and 80 (rather than 80 exactly). To avoid differentially misclassifying the exposure due to changes in assay sensitivity and left-censoring, we set all viral loads at or below 80 to 40 (half of 80); in addition, we also set all viral loads at LLDs other than 80 to 40.

**Figure 2:**
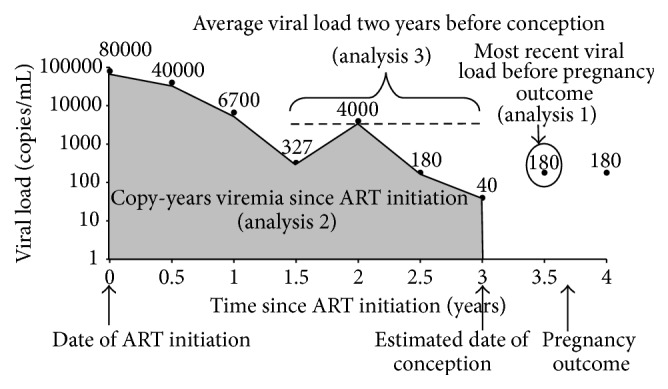
Construction of copy-years viremia, a calculation of the area under the longitudinal viral load curve from ART initiation to estimated date of conception (analysis 2). Also illustrated in the figure are the average viral load two years before conception (analysis 3), equivalent to copy-years viremia in two years before conception, and the most recent viral load measure proximal to pregnancy outcome (analysis 1).

For analysis 1, we used the exposure of most recent viral load before the pregnancy ended (Figure 2). For this analysis, the study population included all HIV-infected women with a viral load measure (whether during or before pregnancy, see Section 2.3) within one year before the pregnancy outcome.

For analysis 2, we used the exposure of log_10_ copy-years viremia from ART initiation to conception. For this analysis, the study population included all HIV-infected women with at least one self-reported pregnancy who initiated ART prior to their pregnancy. This analysis captured long-term ART users and did not include those who only initiated ART during pregnancy (however, these pregnancies were captured in analyses 1 and 3). Ideally, we would have liked to assess lifetime copy-years viremia since seroconversion; however because few WIHS participants seroconverted, these data were unavailable. Left-censoring copy-years viremia at a well-defined point, in this case ART initiation, was the next best option [24, 25]; controlling for CD4 count and other factors at time of ART initiation helped account for potential differences prior to ART initiation. Others have taken this approach and found that log_10_ copy-years viremia since ART initiation was predictive of mortality [24, 25].

Log_10_ copy-years viremia from ART initiation to conception was defined as the area under a patient's longitudinal viral load curve from ART initiation to the estimated date of conception (Figure 2) [23]. Viral loads during pregnancy were not included to prevent differential misclassification by outcome, since women with a live birth would have more exposure time until their pregnancy outcome than a miscarriage or stillbirth. We conducted a complete case analysis, excluding women who did not have a viral load measure within a baseline window from 24 weeks prior to up to four weeks following ART initiation. A vast majority of women meeting this exclusion criterion enrolled in the WIHS after they initiated ART; thus this missing data was assumed to be uninformative.

For analysis 3, we used the exposure log_10_ copy-years viremia in the two years before conception and did not restrict it to ART initiators. For this analysis, we defined the study population as all HIV-infected women with at least two viral loads in the two years before conception. The calculation of this exposure was analogous to that for analysis 2, except this exposure only included those viral load values during the two years before conception (Figure 2). Since viral loads were not measured in the study exactly at conception and two years prior to conception, we imputed viral load values for these dates using the most proximal viral load to that time point. This enabled calculation of log_10_ copy-years viremia in the two years before conception using a homogeneous time frame for each woman.

### 2.2. Statistical Analysis

Demographic and clinical characteristics of women and their pregnancies were described using proportions for categorical variables and medians for continuous variables. Log-binomial and linear regression models were used to estimate the associations between the three exposures and the proportions of total pregnancy losses out of total pregnancies, using generalized estimating equations with an exchangeable correlation structure to account for within-subject correlation (multiple pregnancies per individual). For each analysis, we considered both continuous and categorical specifications of the viral load measures. Categories of cross-sectional viral load measures (analysis 1) were based on clinically informative cut-points (1.60, 3.00, and 4.00 log_10_ viral load), while the categories for cumulative viremia measures (analyses 2 and 3) were based on statistical quartiles since the meaningful cut-points of viremia in this context are unknown.

Multivariable models were used to adjust for covariates identified using posited causal directed acyclic graphs (DAGs). Based on our DAGs, confounders included for adjustment were maternal age, race/ethnicity, previous pregnancy loss, CD4 count, ART use, smoking, and injection and noninjection drug use (IDU and NIDU). CD4 counts and ART use were measured at baseline, which was defined as the beginning of exposure assessment: visit of viral load ascertainment before the pregnancy ended (analysis 1), visit when ART was initiated (analysis 2), and first visit during the two-year time period preceding pregnancy (analysis 3) (Figure 2). Smoking, IDU, and NIDU were measured at the WIHS visit prior to the pregnancy outcome. This measurement occurred during either the woman's pregnancy or the nearest measure to conception, representing the closest proxy to these covariates during pregnancy. We used a linear spline to flexibly control for maternal age. After exploring the frequencies of IDU among study participants (approximately 1-2% in all three populations), we excluded women with IDU from all analyses to reduce potential unmeasured confounding.

ART was controlled for in all analyses, through restriction in analysis 2 and through adjustment for ART use at baseline in analyses 1 and 3. We did not want to control for ART use after baseline, since ART use after the beginning of exposure assessment could be considered a downstream causal effect of viral load burden, and adjustment for it might bias estimates of the total effects of the exposures. Indicators variables were used to categorize ART use as mono-/dual therapy, HAART, or none. We did not adjust for duration of ART because we assumed that there was no independent effect of duration of ART on pregnancy loss external of its effect on our viral load burden.

### 2.3. Sensitivity Analyses

We conducted several analyses to assess the sensitivity of our results to key assumptions. For analyses 1 and 3, we restricted the analyses to pregnancies with exposure during 1998–2013. We altered the inclusion criterion for analysis 2 from ART initiation since 1994 to HAART initiation since 1998. For analyses 1 and 3, we also controlled for HAART instead of any ART. These sensitivity analyses were used to see if capturing slightly more modern study population and ART regimens changed the conclusions of the analyses. Ideally, we would have preferred to restrict the population to a more recent subgroup, but our sample size did not provide enough power for that analysis.

For analysis 1, we only included viral loads that were measured during pregnancy, since the original exposure allowed for measurements prior to pregnancy, as long as they were within one year of the pregnancy outcome. For analysis 2, we dropped the first viral load following ART initiation from the calculation of copy-years viremia to allow time for viral response to therapy. We also separately assessed the results for analysis 2 when adjusting for time on ART, to relax the assumption that the time on ART did not impact the effect of copy-years viremia (see Section 4). For analysis 3, we altered the baseline by varying the exposure period from two years prior to conception to one and three years prior to conception. For each analytic population, stillbirths were excluded from our definition of pregnancy loss to determine if focusing on miscarriages altered our results. Finally, we considered potential effect measure modification by CD4 count and adjustment for CD4 nadir instead of baseline CD4 count.

## 3. Results

### 3.1. Study Population

There were 932 self-reported singleton pregnancies from 514 HIV-infected women enrolled in the WIHS from October 1, 1994, to March 31, 2013 (Figure 1). The population for analysis 1, where the exposure was the most recent viral load before pregnancy outcome, comprised 461 pregnancies among 336 women; 160 (35%) of these pregnancies resulted in losses (148 miscarriages and 12 stillbirths). The population for analysis 2, where the exposure was log_10_ copy-years viremia from ART initiation to conception, comprised 253 pregnancies among 149 women; 93 (37%) of these pregnancies resulted in losses (88 miscarriages and 5 stillbirths). The population for analysis 3, where the exposure was log_10_ copy-years viremia in the two years before conception, comprised 380 pregnancies among 285 women; 151 (40%) of these pregnancies resulted in losses (139 miscarriages and 12 stillbirths).

### 3.2. Population and Pregnancy Characteristics

Most women in the study populations were black and low-income; the median maternal ages were in the early 30s (Table 1). A high percentage of women reported smoking at the visit prior to the pregnancy outcome (35–37% in all three populations). For analysis 1, the median most recent log_10_ viral load was 2.5 (interquartile range (IQR): 1.6, 3.8), with a median time from sample measurement to pregnancy outcome of 13 weeks (IQR: 6, 20). Of the 461 pregnancies eligible for analysis 1, 393 (85%) occurred among women who had ever initiated ART (Table 1). Of those 393 pregnancies, 324 occurred among women who reported currently taking ART at the visit of their viral load assessment, of whom 39 (11%) were on monotherapy, 36 (11%) were on combination therapy, and 249 (73%) were on HAART. Of note, 181 pregnancies included in analysis 1 (39%) had most recent viral load measures at or below the lower limit of detection. Also, for 356 pregnancies (77%) this viral load was measured during their pregnancy (see Section 2.3). For analysis 2, the median log_10_ copy-years viremia from ART initiation to conception was 4.4 (IQR: 3.8, 4.9), with a median time from ART initiation to conception of 4.3 years (IQR: 2.2, 7.5). For analysis 3, the median log_10_ copy-years viremia in the two years before pregnancy was 5.7 (IQR: 4.3, 6.5). Univariate results indicated that prior pregnancy loss, maternal age, smoking, and noninjection drug use were all associated with pregnancy loss (Supplemental Table 1 in Supplementary Material available online at http://dx.doi.org/10.1155/2015/362357).

### 3.3. Effect Estimates

In analysis 1, the adjusted risk ratio (aRR) for the effect of a one-log increase in most recent viral load measure before pregnancy outcome on the risk of pregnancy loss was 1.17 (95% confidence interval (CI): 1.01, 1.35); the corresponding adjusted risk difference (aRD) was 0.04 (95% CI: −0.01, 0.08). Comparisons of categories of most recent viral load further suggest an increasing trend in both relative and absolute risk measures with increasing viral load (Table 2); notably, the absolute adjusted increase in risk was 0.14 (95% CI: −0.01, 0.28) for pregnancies whose viral load measurement was in the highest category (>4.00 log_10_) compared to those women whose final viral load was in the lowest category (≤1.60 log_10_). In analyses 2 and 3, the aRRs for the effect of a one-unit increase in log_10_ copy-years viremia from ART initiation to conception and viremia in the two years before conception on the risk of pregnancy loss were 0.80 (95% CI: 0.69, 0.92) and 1.00 (95% CI: 0.90, 1.11), respectively. The corresponding aRDs were −0.10 (95% CI: −0.14, −0.05) and −0.01 (95% CI: −0.01, 0.03).

Most of the different scenarios explored in the sensitivity analyses did not qualitatively alter the results (Table 3). The aRR for log_10_ copy-years viremia from ART initiation to conception when restricted to HAART initiators since 1998 moved closer to the null (Table 3). There did not appear to be any effect measure modification when comparing the effect measures among those with CD4 greater than 500 to those less than 500 for analyses 1 and 2 (Table 3). For analysis 3, the effect of two-year viremia remained close to the null among those with CD4 below 500, but the aRR for log_10_ copy-years viremia in two years was 1.16 (95% CI: 0.98, 1.36) among those with CD4 greater than 500.

## 4. Discussion

Previous research on the interplay between HIV infection, immune system inflammation, and pregnancy loss is both sparse and contradictory [13–17, 21, 22]. In this study, we found that the most recent viral load before birth outcome was associated with pregnancy loss among HIV-infected women (analysis 1). When controlling for confounding factors (maternal age, race, income, prior pregnancy loss, smoking, CD4 count, and ART use), a 14% absolute increase in risk of pregnancy loss was observed for the highest category compared to the lowest category of viral load. These results confirm the harmful effects of high maternal viral load on birth outcomes that others have observed in observational HIV cohorts [16, 21, 22].

In addition to benchmarking our work against the cross-sectional analyses of other studies, we also assessed two measures of cumulative viral load. The results of analysis 2 counterintuitively suggest a protective effect of higher log_10_ copy-years viremia from ART initiation to conception on pregnancy loss. There is no biological basis for the idea that increased cumulative viremia (and attendant inflammation) is protective against pregnancy loss: this apparently protective result should be interpreted cautiously and may be biased by residual confounding or measurement error. Women who became pregnant with elevated viral loads and higher cumulative viremia may have been treated aggressively with ART to reduce their viral burdens, which could be contributing to the protective effect of higher cumulative viremia. Additionally, if a woman was receiving ART for a longer period of time, she was potentially infected for a longer period of time and thus may have had larger copy-years viremia. In this instance, the protective effect of long-term ART could be outweighing any detrimental effects of cumulative inflammation and contributing to the protective effect we observed with this measure. Another issue that may come into play with this measure is the underlying assumption that the means of accumulating a specific amount of copy-years viremia did not impact the effect of that exposure. For example, we assumed that an exposure of three log_10_ copy-years viremia had the same impact on risk of pregnancy loss whether the exposure was accrued over 10 years, three years, or one year. Other cumulative measures, such as smoking pack-years, have faced similar scrutiny [34, 35], and future studies may wish to explore this issue further. To relax this assumption, we adjusted for time on ART in a sensitivity analysis and the effect estimates were unaffected (Table 3). Overall, analysis 2 indicates that copy-years viremia since ART initiation might not be a useful measure for reproductive outcomes, even though others have illustrated its utility for mortality [24, 25] and lymphoma [26].

In analysis 3, by constructing a time-constrained measure of viremia, we were focusing on comparisons of recent average viral load intensities before conception. The effect of viremia on pregnancy loss was no longer protective and now at the null, suggesting little harmful effect of copy-years viremia in the two years prior to conception. However, the results from our sensitivity analysis considering effect measure modification by CD4 count greater than 500 indicate that there is a potentially harmful effect of viremia in the two years prior to conception among those with higher CD4 counts, but not among those with lower CD4 counts (Table 3). Although viral loads were not measured frequently enough to assess copy-years viremia during pregnancy, future studies may wish to explore this further given our cross-sectional results (analysis 1).

Our analyses had several limitations. First, the study covered a twenty-year period with secular changes over time, particularly in the use of antiretroviral therapy. We attempted to control for this potential lack of homogeneity over the study period by controlling for measured confounders and conducting a sensitivity analysis using only data from the “post-HAART” era, but we cannot discount the possibility of unmeasured confounding biasing our results. This limits any causal interpretations we might want to ascribe to our results. In addition, all pregnancy outcomes were self-reported, which may lead to bias. One particular concern is that some miscarriages might actually have become elective abortions if the pregnancy continued, or elective abortions might have become miscarriages, which would result in misclassification of miscarriage. As with all other retrospective studies on miscarriage, we were limited by the fact that many miscarriages occurring earlier in pregnancy were unrecognized by mothers, further limiting the accuracy of our outcome measurement [36]. Furthermore, the use of pregnancy loss as a composite outcome combines first-trimester miscarriage, late miscarriage, and stillbirth. Although the etiologic risk factors for these separate outcomes may vary, the study is underpowered to investigate these outcomes separately. We did exclude stillbirths in a sensitivity analysis to determine if focusing on miscarriages altered our results and found no differences. Also, while pregnancies were systematically ascertained through self-report at each biannual visit, there is still a possibility of biased detection by patient characteristics if certain individuals were more likely to report their pregnancies than others.

We also acknowledge other limitations specific to the exposures beyond those addressed above. Viral loads were collected semiannually; thus we were forced to assume that these measurements were representative of women's continuous viral load burdens. Another issue with cumulative exposure measures is that we were unable to account for within-person variation. For example, even if two pregnancies have similar cumulative viral load, the patterns of exposure leading to this composite measure are not captured [37]. Furthermore, our analysis provides no information regarding the effect of copy-years viremia on fertility. There is evidence from trials of low-dose aspirin that inflammation plays a role in impairing conception [38]; thus it is plausible that cumulative viremia may be impacting fertility.

Our analyses incorporated high-quality longitudinal measurements of viral load collected prospectively in an established clinical cohort. A main strength of this study is the utilization of two new constructs of viral load burden. As copy-years viremia has only recently been explored as an exposure for various prognoses [23–26], continued research on this exposure is valuable. We further add to the body of literature on HIV viral load burden and pregnancy loss by reporting risk differences in addition to risk ratios.

In conclusion, these results help elucidate the role of viral load burden in pregnancy loss among HIV-infected women. As this is the first study addressing cumulative viral load burden and pregnancy loss, future studies are needed to further investigate these findings. We addressed multiple constructs of viral load burden, including two new approaches to copy-years viremia as well as one cross-sectional measure of viral load for comparison to the literature. While cumulative viral load burden does not appear to be an informative measure for pregnancy loss risk in this setting, cross-sectional viral load proximal to pregnancy was associated with an increased risk of pregnancy loss on both the relative and absolute scales. These results emphasize the importance of early identification of pregnancy, initiation of or adjustment to more appropriate ART during pregnancy, and subsequent control of viral load to potentially reduce the risk of pregnancy loss.

## Supplementary Material

Supplementary Table 1. reports the univariate risk ratios and risk differences of the association between potential risk factors with pregnancy loss among HIV-infected women enrolled in the Women's Interagency HIV Study (WIHS) from October 1,1994 to March 31, 2013 and their respective pregnancies meeting eligibility criteria for analysis 1 (N = 461).

## Figures and Tables

**Table 1:** Characteristics of HIV-infected women enrolled in the Women's Interagency HIV Study (WIHS) from October 1, 1994, to March 31, 2013, and their respective pregnancies meeting eligibility criteria for three analytic populations: (i) most recent viral load before pregnancy outcome, (ii) copy-years viremia since ART initiation, and (iii) copy-years viremia in the two years before conception.

Characteristic	Most recent VL (i)		Viremia since ART (ii)		Viremia in two years (iii)	
	Women (*n* = 336)	Pregnancies (*n* = 461)	Women (*n* = 149)	Pregnancies (*n* = 253)	Women (*n* = 285)	Pregnancies (*n* = 380)
Race						
Black	212 (63)	289 (63)	80 (54)	140 (55)	175 (61)	235 (62)
White	67 (20)	93 (20)	33 (22)	61 (24)	59 (21)	79 (21)
Other^*∗*^	57 (17)	79 (17)	36 (24)	52 (21)	51 (18)	66 (17)
Income/year						
<$12,000	170 (53)	233 (53)	66 (46)	114 (47)	131 (48)	178 (49)
$12,001–$36,000	117 (36)	152 (35)	59 (41)	87 (36)	107 (39)	133 (37)
>$36,000	34 (11)	55 (13)	20 (14)	42 (17)	35 (13)	52 (14)

Pregnancy dependent measures						
CD4 count^†^	—	438 (294–644)	—	369 (242–528)	—	445 (298–661)
IDU^‡^	—	7 (2)	—	3 (1)	—	4 (1)
Prior loss^§^	—	137 (30)	—	80 (32)	—	119 (31)
Smoking	—	166 (36)	—	87 (35)	—	138 (37)
Maternal age	—	32 (29–37)	—	34 (30–38)	—	33 (29–38)
ART initiated^†^	—	393 (85)	—	All	—	225 (59)
NIDU	—	84 (18)	—	36 (14)	—	66 (17)

Categorical variables expressed as number (% total); continuous variables as median (interquartile range). WIHS = Women's Interagency HIV Study, ART = antiretroviral therapy, VL = viral load, IDU = injection drug use, and NIDU = noninjection drug use.

^*∗*^Unknown, Asian, Hispanic, Pacific Islander, Native American, and Alaskan.

^†^Measured at baseline, the visit at which the first viral load measure for each analysis occurred.

^‡^Injection drug users were excluded for the main analysis to reduce confounding.

^§^Self-reported previous miscarriage or stillbirth prior to and during WIHS enrollment.

**Table 2:** Risk ratios and risk differences of the association of pregnancy loss with most recent cross-sectional viral load measure before the pregnancy ended (*N* = 454), log_10_⁡ copy-years viremia from ART initiation to conception (*N* = 250), and log_10_⁡ copy-years viremia in the two years prior to conception (*N* = 376).

	Pregnancy loss	Relative effect measures		Absolute effect measures	
	Losses/*N*	RR (95% CI)	aRR (95% CI)^*∗*^	RD (95% CI)	aRD (95% CI)^*∗*^
Analysis 1: most recent log_10_⁡ viral load measure					
Continuous exposure	158/454	1.27 (1.13, 1.42)	1.17 (1.01, 1.35)	0.10 (0.06, 0.14)	0.04 (−0.01, 0.08)
Dichotomous exposure					
≤1.60	41/181	1.	1.	0.	0.
>1.60	117/273	1.89 (1.39, 2.58)	1.23 (0.87, 1.74)	0.20 (0.11, 0.29)	0.05 (−0.04, 0.14)^†^
Categorical^‡^					
Q1: ≤1.60	41/181	1.	1.	0.	0.
Q2: 1.61–3.00	30/91	1.47 (0.98, 2.19)	1.15 (0.79, 1.68)	0.10 (−0.01, 0.21)	0.03 (−0.06, 0.12)^ †^
Q3: 3.01–4.00	39/90	1.87 (1.27, 2.75)	1.20 (0.77, 1.87)	0.19 (0.07, 0.32)	0.04 (−0.09, 0.17)^ †^
Q4: >4.00	48/92	2.36 (1.68, 3.31)	1.59 (0.99, 2.56)	0.30 (0.18, 0.42)	0.14 (−0.01, 0.28)^ †^

Analysis 2: log_10_⁡ copy-years viremia since ART initiation					
Continuous exposure	92/250	0.83 (0.73, 0.95)	0.80 (0.69, 0.92)	−0.07 (−0.13, −0.02)	−0.10 (−0.14, −0.05)
Quartile of exposure^‡^					
Q1: 0–3.78	29/62	1.	1.	0.	0.
Q2: 3.79–4.38	24/63	0.84 (0.55, 1.30)	0.85 (0.56, 1.30)	−0.07 (−0.25, −0.11)	−0.05 (−0.21, 0.12)
Q3: 4.39–4.94	22/63	0.72 (0.45, 1.14)	0.70 (0.44, 1.13)	−0.13 (−0.31, −0.05)	−0.15 (−0.32, 0.03)
Q4: >4.94	17/62	0.61 (0.37, 1.00)	0.60 (0.36, 0.99)	−0.18 (−0.35, −0.01)	−0.18 (−0.36, −0.01)

Analysis 3: log_10_⁡ copy-years viremia in two years before pregnancy					
Continuous exposure	149/376	1.12 (1.01, 1.24)	1.00 (0.90, 1.11)	0.05 (0.01, 0.09)	−0.01 (−0.05, 0.03)
Quartile of exposure^‡^					
Q1: 0–4.26	28/94	1.	1.	0.	NC
Q2: 4.27–5.73	34/94	1.19 (0.78, 1.81)	0.90 (0.61, 1.32)	0.06 (−0.08, 0.20)	NC
Q3: 5.74–6.49	46/94	1.57 (1.07, 2.29)	1.07 (0.72, 1.57)	0.17 (0.03, 0.31)	NC
Q4: >6.49	41/94	1.43 (0.97, 2.09)	0.92 (0.63, 1.35)	0.13 (−0.01, 0.26)	NC

ART = antiretroviral therapy, RR = risk ratio, aRR = adjusted risk ratio, RD = risk difference, and aRD = adjusted risk difference. NC = nonconvergence of model.

^*∗*^Adjusted for maternal age (with a linear spline at age 35), race, income, noninjection drug use, prior pregnancy loss, smoking, CD4 count, and ART use (separately controlling for mono-/dual and HAART versus none) prior to viral load measurement (for analyses 1 and 3).

^†^Point estimates controlled for any ART instead of separately controlling for mono-/dual and HAART due to model nonconvergence.

^‡^Categories of cross-sectional viral load measures (analysis 1) were based on clinically informative cut-points, while the categories for cumulative viremia measures (analysis 2) were based on statistical quartiles.

**Table 3:** Sensitivity analyses; adjusted^*∗*^ risk ratios for a one-unit increase in exposure.

Sensitivity analysis description	aRR^*∗*^ (95% CI)		
	Most recent VL	Viremia since ART	Viremia in two years
Original	1.17 (1.01, 1.35)	0.80 (0.69, 0.92)	1.00 (0.90, 1.11)
Restricted to exposure after 1998 and adjusted for HAART	1.27 (1.07, 1.50)	0.99 (0.82, 1.17)	0.97 (0.83, 1.13)
Dropped VL at ART initiation	N/A	0.83 (0.68, 1.03)	N/A
Adjusted for time on ART	N/A	0.80 (0.68, 0.95)	N/A
Only included VL during pregnancy	1.10 (0.87, 1.39)	N/A	N/A
Time frame changed to one year	N/A	N/A	0.87 (0.75, 1.00)
Time frame changed to three years	N/A	N/A	0.97 (0.87, 1.09)
Excluded stillbirths	1.18 (1.01, 1.38)	0.80 (0.69, 0.93)	0.99 (0.88, 1.12)
Modification by CD4			
CD4 ≤ 500	1.15 (0.99, 1.33)	0.81 (0.68, 0.97)	0.94 (0.82, 1.07)
CD4 > 500	1.17 (0.95, 1.43)	0.81 (0.67, 0.98)	1.16 (0.98, 1.36)
Adjusted for CD4 nadir	1.18 (1.03, 1.35)	0.79 (0.69, 0.91)	1.02 (0.91, 1.14)

aRR = adjusted risk ratio, ART = antiretroviral therapy, HAART = highly active antiretroviral therapy, and VL = viral load.

^*∗*^Adjusted for maternal age (with a linear spline at age 35), race, income, prior pregnancy loss, smoking, noninjection drug use, CD4 count at baseline, and ART use at baseline (separately controlling for mono-/dual and HAART versus none).
