# Supplementary material for: The Effects of Viral Load Burden on Pregnancy Loss among HIV-Infected Women in the United States
Source: Infect Dis Obstet Gynecol. 2015 Oct 25;2015:362357. doi: 10.1155/2015/362357 (PMC4637076; doi:10.1155/2015/362357)
Supplement: Supplementary file 1 — Supplementary Table 1. reports the univariate risk ratios and risk differences of the association between potential risk factors with pregnancy loss among HIV-infected women enrolled in the Women's Interagency HIV Study (WIHS) from October 1,1994 to March 31, 2013 and their respective pregnancies meeting eligibility criteria for analysis 1 (N = 461). [file 362357.f1.pdf]

Supplemental Table 1. Univariate risk ratios and risk differences of the association between potential risk factors with pregnancy loss among HIV-infected women enrolled in the Women's Interagency HIV Study (WIHS) from October 1,1994 to March 31, 2013 and their respective pregnancies meeting eligibility criteria for analysis 1 (N=461).

	Pregnancy loss	Crude Univariate Associations	
	Losses/N	RR (95% CI)	RD (95% CI)
Race			
White	24/93	1.	0.
Black	110/289	1.42 (0.98, 2.06)	0.11 (0.004, 0.22)
Other*	26/79	1.24 (0.77, 2.01)	0.06 (-0.08, 0.21)
Income/year†			
<$12,000	87/233	1.	0.
$12,001-$36,000	52/152	0.92 (0.69, 1.23)	-0.03 (-0.13, 0.07)
>$36,000	13/55	0.69 (0.42, 1.12)	-0.11 (-0.24, 0.02)
CD4 count†			
CD4≤500	91/265	1.	0.
CD4>500	67/192	1.02 (0.78, 1.33)	0.01 (-0.08, 0.10)
Prior loss§			
No	90/324	1.	0.
Yes	70/137	1.86 (1.48, 2.35)	0.24 (0.15, 0.33)
Current smoking†			
No	66/291	1.	0.
Yes	91/166	2.42 (1.87, 3.13)	0.32 (0.23, 0.41)
Maternal age			
19-29	38/139	1.	0.
30-39	91/258	1.33 (0.98, 1.80)	0.09 (-0.001, 0.17)
40-49	28/60	1.72 (1.16, 2.54)	0.19 (0.04, 0.36)
ART use †			
None	77/136	1.	0.
Mono/dual	19/75	0.43 (0.27, 0.68)	-0.32 (-0.45, -0.18)
HAART‡	64/249	0.46 (0.35, 0.60)	-0.31 (-0.41, -0.20)
NIDU†			
No	111/371	1.	0.
Yes	45/84	1.80 (1.38, 2.35)	0.23 (0.11, 0.35)

WIHS=Women's Interagency HIV Study, ART=antiretroviral therapy, HAART=highly-active antiretroviral therapy, VL=viral load, IDU=Injection drug use, NIDU=Non-injection drug use.
* Includes unknown, Asian, Hispanic, Pacific-Islander, Native American, and Alaskan.
† Measured at the visit prior to the pregnancy outcome.
‡ HAART was defined according to the US Department of Health and Human Services/Kaiser Panel guidelines [29].
§ Self-reported previous miscarriage or stillbirth prior to and during WIHS enrollment.
